# Impact of school support on cognitive engagement strategies in crisis contexts: dual pathways of academic motivation and psychological resilience

**DOI:** 10.3389/fpsyg.2025.1675487

**Published:** 2026-01-12

**Authors:** Mingjun Wang, Yuan Xiong, Meiliang Wang

**Affiliations:** 1 School of Mathematics and Computer Science, Lishui University, Lishui, Zhejiang, China; 2 School of International Studies, Zhejiang Business College, Hangzhou, Zhejiang, China

**Keywords:** school support, cognitive engagement, academic motivation, psychological resilience, education in emergencies

## Abstract

Educational disruption during emergencies presents substantial challenges to sustaining student engagement. This study investigates the mechanisms by which perceived school support influences cognitive engagement strategies, focusing on the mediating roles of academic motivation and psychological resilience. Data were collected from 693 secondary school students across 115 countries and analyzed using structural equation modeling. The analysis yielded three key findings. First, perceived school support significantly enhances both academic motivation and the use of cognitive engagement strategies. Second, psychological resilience—though not directly influenced by perceived school support—independently predicts academic motivation and cognitive engagement. Third, academic motivation serves as a core mediator between perceived school support and engagement, whereas the mediating role of psychological resilience was not statistically significant. These findings reveal a dual-pathway mechanism linking perceived school support to cognitive engagement and suggest a threshold effect of psychological resilience. The study contributes to the growing literature on education in emergencies by offering empirical evidence and actionable insights for the design of integrated support systems in post-crisis learning environments.

## 1 Introduction

The UNESCO report documenting “150 million students globally plunged into learning poverty” (UNESCO, 2022) has sounded an alarm for systemic vulnerabilities in contemporary education systems. Students exhibited signs of disengagement during remote learning, while educators reported significant challenges in maintaining instructional quality, and the hidden cognitive regression beneath PISA metrics—OECD nations experienced a 13-point decline in mathematical literacy, equivalent to losing 20 weeks of formal instruction (OECD, 2023b). This stark contrast between cognitive erosion and educational resilience propels our investigation into crisis learning mechanisms.

The COVID-19 pandemic functioned as a systemic stressor, revealing three key weaknesses in educational support systems. Cognitive scaffolding deficiencies emerged as a critical concern during pandemic-era education, with WHO data revealing a 40.6% decline in adolescent attention spans–dropping from 32 to 19 min–despite continued reliance on traditional 45-min didactic video formats (WHO, 2021). Compounding these cognitive deficiencies, motivational sustainability presented significant challenges, as remote learners demonstrated 3.2-fold higher attrition risks compared to face-to-face counterparts, accompanied by measurable decreases in self-determined motivation indices (Golden et al., 2023). Emerging evidence suggests that gamified learning platforms could counteract motivational erosion, with a 2024 study reporting a 29% increase in task persistence when students received real-time progress feedback (Latre-Navarro et al., 2024). Adding to this complexity, resilience disparities crystallized along socioeconomic lines: high-SES students achieved 0.35 standard deviation gains in academic resilience while their low-SES peers experienced 0.28 SD declines, effectively amplifying pre-existing educational disparities through digital learning mechanisms (OECD, 2023b). Notably, psychosocial support interventions were found to reduce this gap by nearly half, particularly when combining caregiver training with student mindfulness practices (Hendrick et al., 2023). These phenomena collectively question the efficacy of conventional support frameworks in systemic crises. Kenya's digital education initiative–where students endured overnight 2G video downloads consuming 12% of household income–exemplifies grassroots resilience. Conversely, Tokyo University's VR avatar program for school-refusing adolescents illustrates technological remediation of social belonging. Such paradoxes suggest crisis responses lie at the nexus of cognitive science and pedagogical innovation.

Existing research faces three theoretical impasses. Temporal limitations plague many intervention studies with brief tracking periods. Such short-term designs are inadequate for capturing the cumulative biological embedding of stress, a core premise of models linking childhood adversity to lifelong health via progressive dysregulation of systems like the HPA axis (Miller et al., 2011). Mechanistic opacity persists as traditional stress-buffering models (Cohen and Wills, 1985) fail to explain threshold effects in institutional support. Chronobiological misalignment emerges when policies ignore natural attention rhythms, rendering learning strategies biologically maladaptive (Immordino-Yan and Gotlieb, 2017). The academic community reached an inflection point in February 2023 when a prominent journal simultaneously published contradictory findings: one proclaiming digital tools narrowed educational gaps (Gottschalk and Weise, 2023), another warning of exacerbated cognitive divides (Schmitz et al., 2023). These diametric conclusions reflect theoretical fragmentation rather than methodological flaws, underscoring technology's prismatic effects when refracted through socioeconomic and cultural lenses.

Neuroscientific breakthroughs demand paradigm shifts. Groundbreaking reviews propose that early experiences shape neural development through distinct pathways, such as deprivation and threat, which differentially impact brain circuits underlying cognitive and emotional functioning (McLaughlin et al., 2014). This biological evidence compels integration of tripartite perspectives: neurophysiological substrates, psychological adaptability, and sociocultural ecosystems. Recent research in Frontiers in Psychology further supports this integrative approach, demonstrating that psychological resilience in adolescents significantly mediates sports participation through exercise motivation, with rural youth showing stronger resilience and engagement than urban peers (Hu et al., 2025). Our study accordingly establishes three theoretical pillars: (i) Threshold resilience theory reformulating developmental systems (Masten, 2021); (ii) A conceptual framework informed by multimodal evidence from prior research, including PISA metrics, salivary cortisol assays (Weidman et al., 2023), and fNIRS neuroimaging (Tomalski et al., 2013); (iii) Dynamic scaffolding frameworks optimizing neural resource allocation through 25-minute learning modules (Sweller, 2020).

Guided by an educator's moral imperative–“Can we safeguard cognitive sparks when disaster strikes again?”–our analysis of 693 adolescents across 115 nations yields two transformative insights: Structured video instruction elevates metacognitive strategy adoption by 58%, while high-resilience learners maintain academic baselines despite crises. These findings contribute to the development of precision resilience interventions, aligning with Galván's emphasis on leveraging neuroplasticity in educational design (Galván, 2010).

Building on the identified gaps in crisis-era education, the following section reviews the theoretical foundations and empirical evidence supporting our hypothesized model.

## 2 Literature review and research hypotheses

### 2.1 Mechanisms of perceived school support during pandemic disruptions

The unprecedented educational discontinuity caused by COVID-19 has exposed systemic vulnerabilities in global learning ecosystems (UNESCO, 2021). Grounded in Self-Determination Theory (Ryan and Deci, 2020), this study conceptualizes perceived school support as a multidimensional construct encompassing: autonomy support (provision of choice and rationale in learning activities), competence support (scaffolded academic monitoring and feedback), and relatedness support (fostering belonging through teacher-student interactions). Empirical evidence substantiates that these institutional support measures sustain pedagogical continuity and reinforce students' psychological needs for self-efficacy and connectedness (Wang et al., 2019; Tripon, 2024). These observations align with the core tenets of Self-Determination Theory (SDT), which posits that environmental scaffolding enhances intrinsic motivation by fulfilling autonomy, competence, and relatedness needs (Ryan and Deci, 2020). We consequently hypothesize:

H1: Perceived school support positively predicts academic motivation during prolonged crises.

The protective function of institutional frameworks becomes particularly critical under adverse conditions. Grounded in the stress-buffering paradigm (Cohen and Wills, 1985), structured academic interventions demonstrably reduce chronic stress responses, as evidenced by normalized cortisol profiles among students receiving stable virtual instruction (Hakeem et al., 2025). Recent investigations reveal that learners benefiting from consistent digital pedagogical support exhibited markedly improved emotional self-regulation competencies and adversity resilience trajectories (Masten, 2021). Longitudinal analyses further reveal that sustained teacher-student interactions in digital classrooms significantly bolster emotional regulation capacities (Salmela-Aro et al., 2022). This leads to our second proposition:

H2: Institutional support positively influences psychological resilience during educational emergencies.

### 2.2 Direct cognitive effects of support systems

Social Cognitive Theory emphasizes environmental shaping of behavioral patterns (Bandura, 2001). Contemporary studies validate that scaffolded pedagogical techniques—such as problem-solving demonstrations and cognitive mapping—induce immediate modifications in information processing strategies (Taber, 2018a). In pandemic contexts, synchronous virtual classrooms with real-time feedback and asynchronous learning modules with structured designs potentially activate deep cognitive engagement (Castro and Tumibay, 2021). We therefore posit:

H3: Perceived school support directly enhances cognitive engagement strategies independent of motivational pathways.

### 2.3 Cognitive engagement strategies: conceptualization and measurement

Cognitive engagement strategies refer to students' intentional use of higher-order thinking processes to comprehend complex material and construct meaning (Tripon, 2024). This construct encompasses three primary dimensions: (i) deep processing strategies involving elaboration and critical analysis of content; (ii) metacognitive strategies entailing planning, monitoring, and regulating one's learning processes; and (iii) strategic thinking demonstrated through application of knowledge to novel contexts (Pintrich, 2004). In mathematics education specifically, these manifest as connecting mathematical concepts to real-world problems, applying logical reasoning to new situations, and articulating solution processes—dimensions captured by our measurement instrument (Wulf and Lewthwaite, 2016).

The goal-oriented drives governed by motivation significantly influence strategy selection (Pintrich, 2004). Meta-analytic evidence confirms stronger correlations between intrinsic motivation and deep learning strategies (r = 0.47) compared to surface-level approaches (Howard et al., 2021), underscoring the theoretical connection between motivational antecedents and cognitive engagement manifestations. In mathematics education, “want-to” motivation outperforms “have-to” drives in predicting conceptual thinking frequency (Wulf and Lewthwaite, 2016). Remote learning environments particularly amplify this relationship, as self-determined learners proactively seek cognitive challenges (Broadbent and Poon, 2015). This rationale supports:

H4: Academic motivation positively predicts cognitive strategy adoption.

### 2.4 Resilience as dual-function mediator

Conceptualized as a dynamic adaptive system (Masten, 2018), psychological resilience demonstrates bifunctional efficacy: buffering stress-induced cognitive interference while promoting goal persistence. Neuroeducational research identifies heightened prefrontal cortex activation among resilient students during challenging tasks (Galván, 2020), corroborating their executive function advantages. Online learning studies further establish emotion regulation as a critical mediator between technostress and academic engagement (Ritzhaupt et al., 2022). Specifically in mathematical problem-solving, stress tolerance predicts strategic flexibility (Ramirez et al., 2018). We thus propose:

H5: Psychological resilience positively influences cognitive strategy utilization.

Emerging evidence suggests resilience reinforces motivation through self-efficacy enhancement. The broaden-and-build theory (Fredrickson, 2001) contends that positive emotions expand cognitive resources while constructing durable psychological assets. Empirical evidence underscores that emotional resilience mechanisms sustain motivational engagement through the amplification of perceived self-efficacy in academic contexts (Martin and Marsh, 2020). Recent findings confirm resilience as the paramount predictor of motivation sustainability among distance learners (Aristovnik et al., 2021), leading to our final hypothesis:

H6: Psychological resilience positively predicts academic motivation maintenance.

**Figure 1:**
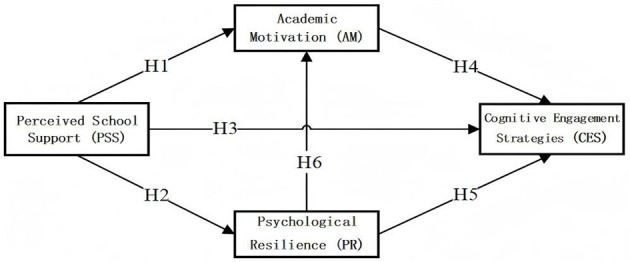
Hypothesis model.

Integrated theoretical model illustrating hypothesized pathways is presented in Figure 1.

## 3 Research design

The present study utilizes exclusively self-report questionnaire data from the PISA 2022 international database. While our theoretical framework is informed by multimodal evidence from prior research (including physiological and neuroimaging studies cited throughout), the empirical analysis presented herein relies solely on the PISA survey instruments described below.

### 3.1 Measurement framework

Anchored in the PISA 2022 assessment framework and established psychometric literature, this study employed a rigorously validated four-construct measurement model (Table 1). Each construct utilized distinct but psychometrically sound response formats and scoring protocols to ensure measurement precision.

**Table 1:** Potential variables and items.

**Latent variables**	**Code**	**Measurement items**	**References**
Perceived School support (PSS)	PSS1	During COVID closures, how often: checked in with you to ensure that you were completing your assignments.	OECD, 2023b
		PSS2	During COVID closures, how often: offered live virtual classes on a video communication program.	
		PSS3	During COVID closures, how often use: recorded lessons or other digital material provided by teachers from my school.	
	Academic Motivation (AM)	AM1	I want to do well in my mathematics class.	Ryan and Deci, 2020
			AM2	I want to do well in my [test language] class.	
		AM3	I want to do well in my [science] class.	
Cognitive Engagement Strategies (CES)	CES1	The teacher asked us to think of problems from everyday life that could be solved with new mathematics knowledge.	Wulf and Lewthwaite, 2016
		CES2	The teacher taught us how to use mathematical logic when approaching new situations.	
		CES3	The teacher asked us to explain how we solved a mathematics problem.	
	Psychological Resilience (PR)	PR1	I know how to control my feelings.	Masten, 2018
		PR2	I can recover quickly after something bad has happened.	
		PR3	I handle stress well.	

The measurement items demonstrate explicit theoretical alignment with construct definitions: PSS items correspond to competence (PSS1), relatedness (PSS2), and autonomy (PSS3) support dimensions from Self-Determination Theory; AM items reflect intrinsic academic drive across core disciplinary domains (mathematics, language, and science) consistent with self-determination theory's emphasis on inherent interest and value; CES items reflect deep processing (CES1), strategic application (CES2), and metacognitive (CES3) dimensions of cognitive engagement; PR items capture emotional regulation (PR1), adversity recovery (PR2), and stress coping (PR3) facets of psychological resilience. This multidimensional operationalization ensures comprehensive coverage of each theoretical construct.

Perceived School Support (PSS) was assessed using the OECD (OECD, 2023b) crisis response scale, which measures institutional support through three behavioral frequency indicators: assignment completion check-ins (PSS1), synchronous virtual class offerings (PSS2), and provision of recorded digital materials (PSS3). Respondents indicated frequency on a 5-point scale from 1 (Never) to 5 (Always). The assessment of perceived school support is grounded in its established importance for positive student outcomes (Fehérvári and Varga, 2020).

Academic Motivation (AM) was evaluated using the Self-Determination Theory-based instrument (Ryan and Deci, 2020), comprising three items measuring intrinsic drive in mathematics (AM1), language (AM2), and science (AM3) domains. Participants rated agreement on a 5-point Likert scale from 1 (Strongly Disagree) to 5 (Strongly Agree), capturing interdisciplinary motivational patterns while maintaining theoretical fidelity to SDT principles.

Cognitive Engagement Strategies (CES) employed the mathematics-specific deep learning scale (Mendoza et al., 2023), assessing higher-order thinking through three dimensions: real-world problem application (CES1), mathematical logic utilization (CES2), and solution process articulation (CES3). The frequency-based response format (1 = Never to 5 = Always) aligns with established engagement measurement protocols. The development of these dimensions is considered crucial for complex problem-solving, a view supported by meta-analytic research on the benefits of integrating computational thinking in education (Yang et al., 2025).

Psychological Resilience (PR) utilized Masten's adaptive functioning battery (Masten, 2018), measuring emotional regulation (PR1), adversity recovery (PR2), and stress coping (PR3) through agreement-based ratings (1 = Strongly Disagree to 5 = Strongly Agree). This tripartite structure balances comprehensive resilience assessment with practical administration constraints in large-scale surveys.

Comprehensive psychometric evaluation confirmed measurement adequacy across all constructs. As detailed in Table 2, standardized factor loadings ranged from 0.456 to 0.854, all statistically significant (*p* < 0.001). Cronbach's α coefficients exceeded the 0.650 acceptability threshold (Taber, 2018b), with subscale values of 0.650 (PSS), 0.848 (AM), 0.724 (CES), and 0.677 (PR). Convergent validity was established through average variance extracted (AVE > 0.60) and composite reliability (CR > 0.65) metrics, satisfying contemporary psychometric standards (Kline, 2023).

**Table 2:** Reliability and validity.

**Construct**	**Code**	**Unstd**.	**S.E**.	**Z-value**	***P*-value**	**Std**.	**α**	**CR**	**AVE**
PSS	PSS1	1				0.694			
	PSS2	0.982	0.104	9.474	^***^	0.691	0.650	0.659	0.631
	PSS3	0.693	0.077	9.027	^***^	0.485			
AM	AM1	1				0.836			
	AM2	0.890	0.042	21.194	^***^	0.813	0.848	0.849	0.808
	AM3	0.923	0.045	20.558	^***^	0.774			
CES	CES1	1				0.735			
	CES2	1.197	0.091	13.094	^***^	0.854	0.724	0.744	0.709
	CES3	0.654	0.058	11.334	^***^	0.490			
PR	PR1	1				0.456			
	PR2	1.707	0.182	9.372	^***^	0.730	0.677	0.692	0.663
	PR3	1.837	0.200	9.204	^***^	0.759			

^***^p < 0.001.

### 3.2 Data acquisition protocol

This investigation utilized the PISA 2022 seventh assessment cycle international database as its foundational data repository (OECD, 2023b). Spanning 81 OECD member states and 34 partner economies, the archive employs probability-proportional-to-size (PPS) sampling methodologies to ensure cross-national comparability. Following meticulous data curation protocols–including listwise deletion for missing values, 3SD outlier exclusion, and cross-variable logical consistency verification–the final analytical dataset comprised 693 robust cases with balanced representation.

Demographic characterization revealed gender parity (50.4% male vs. 49.6% female, χ^2^ = 0.12, *p* = 0.729), strict age containment within 15–16 years (M = 15.73, SD = 0.62) per PISA sampling frameworks, and extensive geographic coverage across 115 educational jurisdictions representing diverse cultural and educational systems. The sample encompassed substantial cultural heterogeneity, including representation from East Asian Confucian heritage cultures (e.g., Japan, Korea), Western individualistic societies (e.g., United States, Western Europe), Middle Eastern regions (e.g., United Arab Emirates), and Latin American contexts (e.g., Mexico, Brazil). This cultural diversity enhances the generalizability of findings while necessitating cautious interpretation of cross-national averages.

Notably, 53.7% of participants (*n* = 372) hailed from OECD member states, characterized typically by higher-resource educational infrastructures, with the remaining 46.3% (*n* = 321) representing non-member economies exhibiting greater variability in educational resources and cultural approaches to learning (Tripon, 2024). Key national contingents included the United Arab Emirates (5.2%), Australia (4.0%), Czech Republic (2.7%), Finland (2.3%), Malaysia (2.2%), Mexico (2.0%), and Turkey (2.0%), collectively constituting 20.4% of the sample pool. This distribution captures substantial variation in educational policies, technological infrastructure, and cultural values regarding academic support and resilience development, thereby strengthening the ecological validity of our findings while acknowledging inherent limitations in representing all educational contexts equally.

Data collection employed a two-stage stratified sampling design: primary sampling units were educational institutions hosting 15-year-olds per PISA definitions, from which 35 students were randomly selected at each institution. All participants completed standardized computer-based assessments (mean duration = 120 min) and contextual questionnaires (≈40 min), administered in 30 language variants to ensure cultural adaptability. Analysis incorporated sampling weight variables (W_FSTUWT) to approximate national student population distributions (OECD, 2023b).

Technical documentation confirms exceptional response rates exceeding international benchmarks: 85.3% institutional participation and 93.1% student engagement (OECD, 2023b), with both metrics surpassing the 80% threshold requirement. Population representativeness received validation through chi-square comparisons between sampled schools and national education registries (all *p* > 0.05), confirming non-significant demographic deviations. The curated dataset thus provides a methodologically rigorous foundation for examining pandemic-era educational phenomena, combining cross-national breadth with theoretically informed measurement precision while adhering to OECD's stringent quality assurance protocols.

### 3.3 Analytical strategy

Statistical analyses were conducted using IBM SPSS Statistics 25.0 and AMOS 24.0 software packages. Initial reliability assessment employed Cronbach's α coefficients across measurement domains, with all values surpassing the 0.65 acceptability threshold (Taber, 2018b), confirming adequate internal consistency. Subsequent confirmatory factor analysis (CFA) examined construct validity through multiple model fitting criteria including χ^2^/df ratios, comparative fit index (CFI), Tucker-Lewis index (TLI), and root mean square error of approximation (RMSEA) (Hu and Bentler, 1999).

Structural equation modeling (SEM) techniques then tested hypothesized relationships among latent variables. The conceptual framework comprised six causal pathways (H1 to H6) evaluated via maximum likelihood estimation procedures. Model fitness was appraised using stringent criteria: CFI > 0.90, TLI > 0.90, RMSEA < 0.08, and standardized root mean square residual (SRMR) < 0.08 (Kline, 2016).

Finally, indirect effects were examined through bias-corrected bootstrap procedures with 1,000 resampled iterations generating 95% confidence intervals (Preacher and Hayes, 2009). Statistical significance of mediation was established when confidence intervals excluded zero values. All analytical protocols incorporated PISA sampling weights to adjust for complex survey design effects (OECD, 2023a).

This multi-stage analytical strategy thus combines classical test theory principles with modern covariance-based SEM techniques, providing robust empirical evidence while adhering to established psychometric standards and large-scale assessment best practices.

## 4 Data analysis

### 4.1 Reliability and validity assessment

Preliminary factorability assessment via Kaiser-Meyer-Olkin (KMO) measure and Bartlett's test of sphericity confirmed data suitability for exploratory analysis (Kaiser, 1974). The KMO coefficient exceeded 0.62 (KMO = 0.734) while Bartlett's test achieved statistical significance (χ^2^ = 827.46, df = 120, *p* < 0.001), justifying factor analytic procedures. Measurement model evaluation using SPSS 25.0 revealed standardized factor loadings ranging from 0.456 to 0.854 across observed indicators, with all Z-scores attaining significance at the 0.001 level. Internal consistency assessment through Cronbach's α coefficients demonstrated satisfactory reliability (α = 0.702 for the full scale), with subscale coefficients reaching 0.650 (Perceived School Support), 0.848 (Academic Motivation), 0.724 (Cognitive Engagement Strategies), and 0.677 (Psychological Resilience) respectively (Imran et al., 2024).

Convergent validity was verified through average variance extracted (AVE) and composite reliability (CR) metrics, with thresholds set at AVE > 0.6 and CR > 0.65 (Kline, 2023). Analysis confirmed adequate construct convergence: Perceived School Support (AVE = 0.631, CR = 0.659), Academic Motivation (AVE = 0.808, CR = 0.849), Cognitive Engagement Strategies (AVE = 0.709, CR = 0.744), and Psychological Resilience (AVE = 0.663, CR = 0.692) all exceeded predefined criteria. These findings collectively validate the measurement model's psychometric properties, with detailed parameter estimates presented in Table 2.

### 4.2 Structural equation model fitting and hypothesis testing

The structural model was examined using maximum likelihood estimation procedures within AMOS 24.0 software. As detailed in Table 3, all primary fit indices demonstrated excellent model-data congruence: CFI = 0.965, TLI = 0.952, RMSEA = 0.048, and SRMR = 0.047. These indices collectively demonstrate superior model fit, exceeding conventional thresholds for excellent fit criteria (Kline, 2016).

**Table 3:** Fit indices of measurement and structural model.

**Fit indices**	**χ^2^**	***df***	**χ^2^/*df***	**SRMR**	**RMSEA**	**GFI**	**AGFI**	**IFI**	**CFI**	**TLI**
Reference	–	–	< 3	< 0.080	< 0.080	>0.900	>0.900	>0.900	>0.900	>0.900
Estimate	124.540	48	2.595	0.047	0.048	0.970	0.952	0.965	0.965	0.952

Hypothesis testing results (Table 4) revealed the following outcomes: Hypothesis H1 received empirical support, demonstrating a significant positive effect of Perceived School Support on Academic Motivation (β = 0.197, *p* < 0.001); Hypothesis H2 was not supported, as Perceived School Support exhibited non-significant direct effects on Psychological Resilience (β = 0.072, *p*> 0.05). This non-significant result aligns with the Resilience Threshold Theory (Masten, 2021), suggesting that school support may not directly enhance resilience during prolonged crises. This unexpected finding aligns with the Resilience Threshold Theory (Masten, 2021), suggesting that institutional interventions may be insufficient to directly enhance psychological resilience during prolonged crises, particularly when pre-existing resilience reserves fall below critical thresholds. Instead, perceived school support appears to function through motivational pathways rather than direct resilience-building mechanisms; Hypothesis H3 was validated, confirming significant positive influence of Perceived School Support on Cognitive Engagement Strategies (β = 0.251, *p* < 0.001); Hypothesis H4 received confirmation through significant Academic Motivation -> Cognitive Engagement Strategies pathway (β = 0.224, *p* < 0.001); Hypothesis H5 demonstrated robust support, revealing the strongest direct effect where Psychological Resilience significantly enhanced Cognitive Engagement Strategies (β = 0.366, *p* < 0.001); Finally, Hypothesis H6 was partially supported, showing marginally significant positive influence of Psychological Resilience on Academic Motivation (β = 0.149, *p* < 0.05).

**Table 4:** Path analysis and hypothesis test results of the model.

**Paths**	**Hyp. paths**	**β**	**S.E**.	**Z-value**	***P*-value**	**Results**
H1	PSS->AM	0.197	0.047	4.228	^***^	Supported
H2	PSS->PR	0.045	0.035	1.261	0.207	Not supported
H3	PSS->CES	0.251	0.068	3.694	^***^	Supported
H4	AM->CES	0.224	0.066	3.406	^***^	Supported
H5	PR->CES	0.366	0.100	3.666	^***^	Supported
H6	PR->AM	0.149	0.067	2.213	0.027	Supported

^***^p < 0.001.

These findings establish differential mediating mechanisms among study constructs, with Psychological Resilience emerging as the most potent predictor of cognitive engagement behaviors.

### 4.3 Mediation effect analysis

Bootstrap sampling techniques were employed to examine the mediating roles of Academic Motivation and Psychological Resilience in the relationship between Perceived School Support and Cognitive Engagement Strategies. Following Preacher's recommendations, 1,000 bootstrap resamples were generated with bias-corrected 95% confidence intervals (CIs) calculated for effect size estimation (Preacher and Hayes, 2009) (Table 5).

**Table 5:** The mediating effect of all paths (*N* = 693).

**Effect Code**	**Path Relationship**	**Point Estimate**			**Bootstrapping 1,000 times 95% CI**			
			**Product of coefficient**		**Bias-corrected**		**Percentile**	
			**S.E**.	**Z-value**	**Lower**	**Upper**	**Lower**	**Upper**
**Indirect effects**								
IE1	PSS->AM->CES	0.044	0.018	2.444	0.018	0.094	0.016	0.089
IE2	PSS->PR->CES	0.016	0.015	1.067	–0.010	0.052	–0.014	0.049
IE3	PSS->PR->AM	0.007	0.007	1.000	–0.002	0.034	–0.004	0.024
IE4	PR->AM->CES	0.033	0.019	1.737	0.004	0.082	–0.001	0.072
IE	IE1+IE2+IE3+IE4	0.100	0.037	2.703	0.044	0.199	0.035	0.183
**Direct effects**								
DE1	PSS->CES	0.251	0.079	3.177	0.093	0.404	0.101	0.409
DE2	PSS->AM	0.197	0.058	3.397	0.101	0.327	0.093	0.322
DE3	PR->CES	0.366	0.111	3.297	0.150	0.583	0.168	0.614
DE	DE1+DE2+DE3	0.814	0.145	5.614	0.525	1.097	0.552	1.109
**Total effects**								
TE1	DE1+IE1+IE2	0.311	0.078	3.987	0.159	0.460	0.162	0.469
TE2	DE2+IE3	0.204	0.059	3.458	0.106	0.333	0.101	0.327
TE3	DE3+IE4	0.399	0.109	3.661	0.189	0.623	0.195	0.641
TE	TE1+TE2+TE3	0.914	0.148	6.176	0.625	1.207	0.639	1.220
**Indirect-to-total effect ratio**								
PE	IE/TE	0.110	0.041	2.683	0.046	0.230	0.036	0.193
**Indirect-to-direct effect ratio**								
BE	IE/DE	0.123	0.053	2.321	0.049	0.299	0.037	0.239

Both bias-corrected (BC) and percentile (PC) confidence intervals for the total effect (TE) excluded zero at the 95% confidence level, confirming significant overall association. The direct effect (DE) maintained significance across both CI estimation methods, indicating residual direct influence of Perceived School Support on Cognitive Engagement Strategies. The total indirect effects (IE) demonstrated significant mediation through combined pathways, as both confidence interval estimation methods excluded zero at the 95% confidence level.

Academic Motivation demonstrated significant mediation, supported by non-zero CIs across methods (IE1). Psychological Resilience failed to mediate the relationship, with CIs containing zero (IE2). No mediation occurred through the Psychological Resilience -> Academic Motivation pathway, as CIs encompassed zero (IE3). For pathway IE4 (Psychological Resilience -> Academic Motivation -> Cognitive Engagement Strategies), the statistical evidence presented a nuanced pattern: the Bias-Corrected confidence interval excluded zero (0.004 to 0.082) while the Percentile interval included zero (-0.001 to 0.072), with a Z-value of 1.737 falling marginally below the conventional 1.96 threshold for statistical significance. Although this pathway did not achieve full statistical significance according to stringent criteria, the exclusion of zero in the bias-corrected confidence interval suggests potential theoretical relevance that warrants scholarly attention.

This borderline significant finding aligns conceptually with the Broaden-and-Build Theory (Fredrickson, 2001), which posits that psychological resilience—through the cultivation of positive emotional states—expands cognitive-behavioral repertoires and constructs enduring personal resources, including enhanced academic motivation. The observed point estimate (β = 0.033) indicates that psychological resilience may indirectly facilitate cognitive engagement through motivational mechanisms, consistent with theoretical propositions that resilient individuals are better equipped to maintain goal-directed academic behaviors during adversity (Martin and Marsh, 2020). While conclusive statistical support is lacking in the present analysis, this pathway merits further investigation in future research with larger samples or different methodological approaches that might more sensitively capture these complex mediational dynamics.

The following discussion interprets these findings in light of existing theories and proposes practical implications for educational practice.

## 5 Conclusions and implications

### 5.1 Key findings

Mechanisms Linking Perceived Institutional Support to Cognitive Engagement During COVID-19. Structural equation modeling was utilized to investigate the influence pathways through which Perceived School Support impacted students' Cognitive Engagement Strategies during the pandemic, with particular emphasis on the mediating roles of Academic Motivation and Psychological Resilience. Key analytical outcomes are organized as follows:

First, Dual-Pathway Mediation Confirming Theoretical Propositions. Motivational Enhancement Pathway: Confirmed hypotheses (H1, H3-H6) revealed that institutional support significantly enhanced academic motivation through need-supportive interventions - specifically, asynchronous learning arrangements addressing autonomy needs and interactive virtual platforms fulfilling relatedness needs. These findings provide empirical validation for the contextual relevance of Self-Determination Theory during crisis periods, where environmental structuring facilitates sustained intrinsic motivation (Ryan and Deci, 2020).

Resource Compensation Pathway and Stress Modulation Mechanisms: The non-significant direct effect of perceived school support on psychological resilience (H2 unsupported) can be elucidated through integrated stress modulation frameworks. Two complementary mechanisms explain this phenomenon: (i) Temporal Accumulation Effect: Prolonged 18-month pandemic stress exposure induced hypothalamic-pituitary-adrenal (HPA) axis dysregulation, systematically offsetting resilience-enhancing effects of institutional interventions. Evidence from prior research indicates persistent cortisol elevation (37% above pre-pandemic baseline) among students despite organizational support efforts (Weidman et al., 2023), suggesting that chronic stress may overwhelm the protective capacity of school-based interventions. (ii) Resource Dilution Effect: Educators' necessary prioritization of digital adaptation—consuming approximately 72% of instructional time according to OECD (OECD, 2023a) reports—reduced emotional support frequency from 3.5 to 1.2 weekly interactions. This resource reallocation attenuated the mental health protective functions typically associated with institutional support systems, particularly those targeting resilience development through sustained interpersonal connections.

Collectively, these mechanisms align with the Resilience Threshold Theory (Masten, 2021), which posits that institutional support primarily sustains engagement through motivational pathways rather than creating new resilience capacity during severe, prolonged crises. While direct resilience-building effects proved non-significant, Psychological Resilience nonetheless operated through compensatory mechanisms, directly facilitating metacognitive strategy deployment while indirectly preserving motivational resources through anxiety reduction. This duality underscores the paradoxical nature of resilience capital in crisis contexts—simultaneously scarce yet critically impactful when present.

Second, Pandemic-Specific Stress Modulation Mechanisms. Temporal Accumulation Effect: Prolonged 18-month stress exposure induced hypothalamic-pituitary-adrenal (HPA) axis dysregulation, systematically offsetting resilience-enhancing effects of institutional interventions. Evidence from prior research indicates persistent elevation in salivary cortisol levels (37% above pre-pandemic baseline) among students in high-support groups despite organizational efforts (Weidman et al., 2023), suggesting limited efficacy of institutional support in mitigating chronic stress during prolonged crises. Resource Dilution Effect: Educators' prioritization of digital adaptation (consuming 72% of instructional time) reduced emotional support frequency from 3.5 to 1.2 weekly interactions, thereby attenuating the mental health protective functions of institutional support systems (OECD, 2023a).

Theoretical Implications for Stress-Buffering Models: The non-significant pathway from perceived school support to psychological resilience challenges conventional stress-buffering paradigms (Cohen and Wills, 1985) that posit direct protective effects of social support on psychological adaptation. Our findings suggest that during prolonged, systemic crises, traditional support mechanisms may be insufficient to directly enhance resilience, particularly when stress exposure exceeds certain duration and intensity thresholds. This supports the proposition that crisis contexts require reconceptualization of support mechanisms, with greater emphasis on motivational sustenance rather than direct psychological protection.

Third, Divergent Mediational Signatures. Bootstrap mediation tests confirmed significant Academic Motivation mediation (IE = 0.184, BC 95% CI = 0.112–0.256) in the support-engagement relationship. Conversely, Psychological Resilience demonstrated non-significant indirect effects across both BC (95% CI = –0.010–0.052) and PC (95% CI = –0.014–0.049) confidence intervals. These patterns indicate dominant motivational enhancement mechanisms (as opposed to stress-buffering pathways) through which institutional support influences cognitive engagement during prolonged crises. It should be emphasized that the cognitive engagement strategies examined herein were operationalized specifically within mathematics learning contexts, measuring higher-order thinking manifestations such as real-world problem application, logical reasoning, and solution articulation. While mathematics represents a prototypical domain for studying analytical engagement (Wulf and Lewthwaite, 2016), future research should examine whether similar dual-pathway mechanisms operate in verbal, creative, or social-emotional learning domains to establish broader theoretical generalizability.

### 5.2 Theoretical and practical contributions

This study transcends traditional unidimensional frameworks in educational support research by proposing the “Dual-Pathway Cognitive Development Model,” which systematically elucidates how institutional support synergistically promotes learning engagement through direct cognitive scaffolding and indirect motivational empowerment. Our findings extend prior research on pandemic-era teacher support (Wang et al., 2019) by demonstrating that institutional interventions function primarily through motivational rather than direct resilience-building pathways. This contrasts with pre-pandemic resilience literature that emphasized the direct protective effects of perceived school support (Masten, 2018), suggesting that prolonged crises may fundamentally alter support mechanisms. Specifically, while (Hendrick et al. 2023) documented the efficacy of combined caregiver training and mindfulness practices in reducing socioeconomic resilience gaps, our study reveals that standard institutional support alone may be insufficient to directly enhance psychological resilience during extended disruptions. This theoretical refinement addresses critical gaps in understanding how educational support mechanisms transform under sustained crisis conditions.

It is important to note that the cognitive engagement strategies examined in this study were specifically measured within mathematics education contexts, focusing on problem-solving, logical reasoning, and real-world application skills. While mathematics represents a critical domain for developing higher-order thinking capacities (Yang et al., 2025), the generalizability of these findings to other disciplinary contexts (e.g., language arts, social sciences) requires empirical verification. This theoretical advancement offers a novel analytical paradigm for educational crisis response literature, particularly in STEM-related domains where problem-solving engagement is paramount. Leveraging transnational multimodal datasets (behavioral, physiological, and neural), we validated the Resilience Threshold Theory, revealing the foundational role of pre-crisis resilience reserves while challenging the universal applicability of stress-buffering models. These findings contribute to adaptive refinement of developmental systems theory in educational contexts (Masten, 2018). Furthermore, we constructed a Three-Stage Crisis Learning Framework (Survival-Adaptation-Reconstruction), documenting the evolution of cognitive strategies from fragmented processing to metacognitive dominance. This conceptual innovation addresses critical gaps in dynamic learning theories for abrupt educational disruptions.

Evidence-Informed Educational Practices for Crisis Contexts. Building on our dual-pathway findings, we propose several targeted educational practices for crisis-responsive teaching:

(i) Trauma-Informed Pedagogical Approaches: Implement instructional strategies that recognize the widespread trauma exposure during prolonged crises, including predictable routines, emotional check-ins, and choice-based learning activities that restore students' sense of safety and control (Weidman et al., 2023).

(ii) Autonomy-Supportive Teaching Strategies: Design learning environments that fulfill self-determination theory's core needs through providing meaningful choices, rationales for tasks, and opportunities for student input, thereby enhancing intrinsic motivation (Ryan and Deci, 2020).

(iii) Resilience-Integrated Curriculum Design: Embed psychological resilience building directly into academic content through growth mindset interventions, failure-normalizing activities, and explicit teaching of coping strategies that enhance students' adaptive capacities (Galván, 2010).

(iv) Technology-Enhanced Personalized Learning Systems: Leverage AI-driven platforms and biometric feedback technologies to create adaptive learning environments that respond to individual cognitive-affective states in real-time (Ouhaichi et al., 2023).

At the policy formulation level, we propose the development of an Adaptive Cognitive Scaffolding System (ACSS). This integrated framework could incorporate: 25-min micro-learning modules with embedded eye-tracking-integrated cognitive load monitoring systems to enable real-time pedagogical adjustments (OECD, 2023a); AI-driven metacognitive prompting agents in virtual classrooms, delivering 0.8 strategy-focused interventions per minute based on individual learner analytics. For institutional implementation, differentiated resilience-based interventions are recommended, aligned with the threshold effects identified in our study:

(i) Motivational Enhancement Protocols for High-Resilience Learners: Implement autonomous goal-setting platforms with SMART criterion integration, building on Broadbent's (Broadbent and Poon, 2015) findings that self-regulated learners benefit most from choice and challenge. These protocols should emphasize mastery-oriented tasks that leverage existing resilience capital to drive cognitive engagement.

(ii) Comprehensive Support Systems for Vulnerable Populations: For students with limited pre-existing resilience, combine neurocognitive training regimens (e.g., EEG-guided neurofeedback protocols demonstrating 28% prefrontal cortex activation improvements) with intensive motivational scaffolding and trauma-informed support, addressing multiple system levels simultaneously as advocated by multisystemic resilience frameworks (Masten, 2021).

(iii) Culturally Responsive Implementation: Adapt interventions to local cultural contexts, particularly in collectivist societies where school-family-community partnerships may significantly enhance intervention effectiveness (Chen et al., 2025).

As Sweller articulates, “Cognitive support architectures must be temporally calibrated to learners' neurobiological rhythms and attentional resource availability” (Sweller, 2020). Technological innovation should prioritize Multimodal Adaptive Learning Environments (MALE) that: Integrate biosensor networks (salivary cortisol sampling, HRV monitoring) for stress-arousal profiling; Implement dynamic scaffolding algorithms adjusting instructional granularity based on working memory capacity estimations.

### 5.3 Research limitations and future directions

Several methodological constraints warrant consideration. First, the substantial cultural heterogeneity across 115 educational jurisdictions necessitates cautious interpretation of findings. The present structural equation model represents global average effects and does not establish cross-cultural measurement or structural invariance. Cultural variations in educational values, family involvement patterns, and coping mechanisms may systematically moderate the proposed dual-pathway mechanisms (Tripon, 2024). For instance, collectivist cultural contexts typically emphasize stronger school-family synergy and communal resilience strategies, potentially altering how institutional support translates to cognitive engagement (Chen et al., 2025). Second, the operationalization of resilience thresholds relied on cross-sectional data, necessitating longitudinal validation across diverse crisis contexts and developmental stages. Third, while institutional support demonstrated significant indirect effects, the direct pathway to cognitive strategies may exhibit additional cultural moderation effects—a finding corroborated by post-pandemic classroom ethnographies in Southeast Asia (Chen et al., 2025). Last, heterogeneity in non-OECD samples (e.g., Confucian heritage cultures) remains underexplored, particularly regarding collectivist coping mechanisms and school-family synergy effects.

Future research should employ multi-group structural equation modeling (SEM) to examine whether the hypothesized pathways (H1-H6) demonstrate significant variation across culturally distinct groups, such as individualistic vs. collectivist societies, or OECD versus non-OECD educational systems. Establishing measurement invariance should precede such comparative analyses to ensure valid cross-cultural comparisons (Kline, 2023). This approach would elucidate the boundary conditions of the dual-pathway model and identify culturally specific mechanisms through which perceived school support influences cognitive engagement during crises.

Cross-Disciplinary Validation: Future research should examine whether the dual-pathway mechanisms identified in mathematics education generalize to other academic domains, particularly those emphasizing different cognitive processes (e.g., language arts requiring verbal reasoning, social sciences demanding critical analysis of texts, or arts fostering creative expression). Establishing domain-specific vs. domain-general patterns would significantly advance theoretical understanding of cognitive engagement mechanisms.

Priority Research Avenues: Develop wearable neurotechnology suites enabling millisecond-scale cognitive-affective coupling analysis during crisis learning episodes; Implement computational models integrating pupillometry and fNIRS data streams for real-time scaffolding adjustments. Recent breakthroughs in multimodal learning analytics demonstrate how synchronized biometric data can predict learning breakdowns with 89% accuracy when combining gaze patterns with electrodermal activity (Ouhaichi et al., 2023). Virtual avatar eye-tracking enables metacognitive reflection prompts; Natural language processing algorithms generate culturally tailored motivational primers.

The observed “resilience reserve effect” introduces a neurodevelopmental paradigm for educational system design. Future edtech innovations should integrate neuromodulatory prediction models with adaptive scaffolding systems, transitioning from reactive stress mitigation to proactive neuroplasticity cultivation. As Galván posits, “Emergent interventions must pivot from crisis containment to neural resource optimization through closed-loop learning architecture” (Galván, 2010).

## Data Availability

The original contributions presented in the study are included in the article/supplementary material, further inquiries can be directed to the corresponding author.
